# Supplementary material for: Expression Profiling and MicroRNA Regulatory Networks of Homeobox Family Genes in Sugarcane Saccharum spontaneum L
Source: Int J Mol Sci. 2022 Aug 5;23(15):8724. doi: 10.3390/ijms23158724 (PMC9369071; doi:10.3390/ijms23158724)
Supplement: Supplementary file 1 [file ijms-23-08724-s001.zip › Supplementary file 14.pdf]

```
               10        20        30        40        50        60        70        80        90
1      ATGGCGATGGTGGTGGTCGGGGGCGGCAAGGACCGGTCCTCGCCTGGCGGCGGTGGGGCGCCGCAGGTGGACACGGGCAAGTACGTGCGCTACACC
1       M  A  M  V  V  V  G  G  G  K  D  R  S  S  P  G  G  G  G  A  P  Q  V  D  T  G  K  Y  V  R  Y  T

              106       116       126       136       146       156       166       176       186
97     CCCGAGCAGGTGGAGGCGCTCGAGCGGGTCTACAGCGAGTGCCCCAAGCCCAGCTCGCTGCGCAGGCAGCAGCTCATCAGGGAGTGCCCCATACTC
33      P  E  Q  V  E  A  L  E  R  V  Y  S  E  C  P  K  P  S  S  L  R  R  Q  Q  L  I  R  E  C  P  I  L

              202       212       222       232       242       252       262       272       282
193    AGCAACATCGAGCCCAAGCAGATCAAGGTCTGGTTCCAGAACCGCAGGTGCCGCGAGAAGCAGCGGAAGGAGGCCTCGCGCCTGCAGACTGTGAAC
65      S  N  I  E  P  K  Q  I  K  V  W  F  Q  N  R  R  C  R  E  K  Q  R  K  E  A  S  R  L  Q  T  V  N

              298       308       318       328       338       348       358       368       378
289    CGGAAGCTGACTGCGATGAACAAGCTGTTGATGGAGGAGAATGACAGGCTGCAGAAGCAGGTCTCCCGACTCGTCTACGAGAATGGGTACATGCGA
97      R  K  L  T  A  M  N  K  L  L  M  E  E  N  D  R  L  Q  K  Q  V  S  R  L  V  Y  E  N  G  Y  M  R

              394       404       414       424       434       444       454       464       474
385    CAGCAGCTCCATAATCCTTCTGCTGCGACCACAGACACGAGCTGTGAGTCTGTGGTCACAAGTGGTCAGCACCACCAACAGCAAAACCCAGCAGCT
129     Q  Q  L  H  N  P  S  A  A  T  T  D  T  S  C  E  S  V  V  T  S  G  Q  H  H  Q  Q  Q  N  P  A  A

              490       500       510       520       530       540       550       560       570
481    CCGCGTCCTCAACGGGACGCGAATAACCCAGCTGGTCTACTAGCTATCGCTGAGGAGACCTTGGCAGAGTTCCTGTCGAAAGCGACAGGAACTGCT
161     P  R  P  Q  R  D  A  N  N  P  A  G  L  L  A  I  A  E  E  T  L  A  E  F  L  S  K  A  T  G  T  A

              586       596       606       616       626       636       646       656       666
577    GTCGATTGGGTGCAAATGGTTGGGATGAAGCCTGGTCCGGATTCCATTGGAATCATCGCTGTTTCGCACAATTGTAGTGGCGTAGCAGCCCGAGCT
193     V  D  W  V  Q  M  V  G  M  K  P  G  P  D  S  I  G  I  I  A  V  S  H  N  C  S  G  V  A  A  R  A

              682       692       702       712       722       732       742       752       762
673    TGCGGCCTAGTGAGCCTTGAGCCCACAAAGGTCGCGGAGATCCTCAAGGATCGCCCTTCTTGGTACCGCGACTGCCGGTGCGTGGATATCCTCCAT
225     C  G  L  V  S  L  E  P  T  K  V  A  E  I  L  K  D  R  P  S  W  Y  R  D  C  R  C  V  D  I  L  H

              778       788       798       808       818       828       838       848       858
769    GTTATCCCTACGGGTAACGGTGGAACTATCGAGCTTATCTACATGCAGACTTACGCACCGACAACTCTGGCGGCACCACGCGACTTTTGGACTCTC
257     V  I  P  T  G  N  G  G  T  I  E  L  I  Y  M  Q  T  Y  A  P  T  T  L  A  A  P  R  D  F  W  T  L

              874       884       894       904       914       924       934       944       954
865    CGATACACTAGTGGTCTTGAGGATGGCAGTCTTGTGATCTGTGAGAGATCATTGACACAATCGACTGGAGGCCCATCAGGACCAAACACTCCAAAT
289     R  Y  T  S  G  L  E  D  G  S  L  V  I  C  E  R  S  L  T  Q  S  T  G  G  P  S  G  P  N  T  P  N

              970       980       990      1000      1010      1020      1030      1040      1050
961    TTTGTCAGAGCCGAAGTGCTTCCTAGTGGCTATTTGATTCGACCTTGTGAGGGAGGTGGCTCCATGATTCACATTGTGGATCATGTTGATTTAGAT
321     F  V  R  A  E  V  L  P  S  G  Y  L  I  R  P  C  E  G  G  G  S  M  I  H  I  V  D  H  V  D  L  D

             1066      1076      1086      1096      1106      1116      1126      1136      1146
1057   GCTTGGAGTGTGCCTGAGGTCCTTAGACCACTTTATGAATCTCCAAAGATTCTTGCACAGAAAACAACTATTGCTGCACTGCGCCACATTAGGCAA
353     A  W  S  V  P  E  V  L  R  P  L  Y  E  S  P  K  I  L  A  Q  K  T  T  I  A  A  L  R  H  I  R  Q

             1162      1172      1182      1192      1202      1212      1222      1232      1242
1153   ATTGCGCATGAATCGAGTGGGGAAATGCCCTATGGAGGGGGCCGCCAGCCAGCGGTGTTGAGAACATTCAGCCAGAGGCTTAGCAGGGGTTTCAAT
385     I  A  H  E  S  S  G  E  M  P  Y  G  G  G  R  Q  P  A  V  L  R  T  F  S  Q  R  L  S  R  G  F  N

             1258      1268      1278      1288      1298      1308      1318      1328      1338
1249   GATGCTGTCAATGGGTTTCCAGATGACGGCTGGTCACTGATGAGCAGTGACGGTGCTGAGGATGTTACTATTGCTATCAACTCCTCTCCAAACAAA
417     D  A  V  N  G  F  P  D  D  G  W  S  L  M  S  S  D  G  A  E  D  V  T  I  A  I  N  S  S  P  N  K

             1354      1364      1374      1384      1394      1404      1414      1424      1434
1345   CTTATTGGTTCTCATGTCAACTCCTCCCAGCTGTTTACTGCGATTGGAGGTGGCATCCTGTGTGCTAAGGCATCAATGCTGCTTCAGAATGTGCCA
449     L  I  G  S  H  V  N  S  S  Q  L  F  T  A  I  G  G  G  I  L  C  A  K  A  S  M  L  L  Q  N  V  P

             1450      1460      1470      1480      1490      1500      1510      1520      1530
1441   CCTGCCCTACTAGTACGATTTCTGAGGGAGCATCGCTCCGAATGGGCTGATCCTGGTGTTGATGCTTATTCTGCTGCTGCTCTGAGGGCTAGTCCA
481     P  A  L  L  V  R  F  L  R  E  H  R  S  E  W  A  D  P  G  V  D  A  Y  S  A  A  A  L  R  A  S  P

             1546      1556      1566      1576      1586      1596      1606      1616      1626
1537   TATGCAGTTCCTGGCTTGCGCGCTAGTGGGTTTATGGGCAGCCAGGTTATTCTACCGCTTGCACATACCTTAGAGAATGAAGAGTTCTTGGAGGTT
513     Y  A  V  P  G  L  R  A  S  G  F  M  G  S  Q  V  I  L  P  L  A  H  T  L  E  N  E  E  F  L  E  V

             1642      1652      1662      1672      1682      1692      1702      1712      1722
1633   ATTAGGCTTGAGGGACACAGCCTCTGCCATGATGAAGTTGTTCTGTCACGAGATATGTATCTTCTGCAGTTGTGCAGTGGCGTGGATGAAAATGCA
545     I  R  L  E  G  H  S  L  C  H  D  E  V  V  L  S  R  D  M  Y  L  L  Q  L  C  S  G  V  D  E  N  A

             1738      1748      1758      1768      1778      1788      1798      1808      1818
1729   GCTGGTGCATGTGCACAGCTTGTCTTTGCACCCATCGATGAATCTTTTGCTGATGATGCACCACTGCTACCCTCAGGCTTCCGTGTCATTCCACTG
577     A  G  A  C  A  Q  L  V  F  A  P  I  D  E  S  F  A  D  D  A  P  L  L  P  S  G  F  R  V  I  P  L

             1834      1844      1854      1864      1874      1884      1894      1904      1914
1825   GACGCAAAGACGGATCCACCATCAGGCACACGCACACTTGACATAGCATCTACTCTTGAGGTTGGATCTGGTGGGACTACTCGTGTTTCAAGTGAT
609     D  A  K  T  D  P  P  S  G  T  R  T  L  D  I  A  S  T  L  E  V  G  S  G  G  T  T  R  V  S  S  D

             1930      1940      1950      1960      1970      1980      1990      2000      2010
1921   GCCTCCAGCACCTGCAACACAAGATCAGTGCTGACCATTGCTTTCCAGTTCTCATATGAGAATCACCTACGGGAAAGCGTCGCAGCAATGGCCAGG
641     A  S  S  T  C  N  T  R  S  V  L  T  I  A  F  Q  F  S  Y  E  N  H  L  R  E  S  V  A  A  M  A  R

             2026      2036      2046      2056      2066      2076      2086      2096      2106
2017   CAATATGTCAGGACTGTGGTGGCATCGGTGCAGAGGGTGGCCATGGCAATAGCTCCTTCCCGTCTTGGTGGACAGCTTGAAATGAAGCAAACTCCA
673     Q  Y  V  R  T  V  V  A  S  V  Q  R  V  A  M  A  I  A  P  S  R  L  G  G  Q  L  E  M  K  Q  T  P

             2122      2132      2142      2152      2162      2172      2182      2192      2202
2113   GGATCTCCTGAGGCACACACACTTGCAAGGTGGATCGGCAGGAGCTACAGGTTTCACACTGGAGCGGAACTCCTTCGCACAGACACCCAATGCACT
```

```
705       G  S  P  E  A  H  T  L  A  R  W  I  G  R  S  Y  R  F  H  T  G  A  E  L  L  R  T  D  T  Q  C  T

                 2218      2228      2238      2248      2258      2268      2278      2288      2298
2209     GATGCTTCCTTGAAAGCACTGTGGCAACACTCAGACTCGATCATGTGCTGTTCCCTGAAGGCTACTCCTGTGTTCACCTTCGCCAACCAAGCCGGC
737       D  A  S  L  K  A  L  W  Q  H  S  D  S  I  M  C  C  S  L  K  A  T  P  V  F  T  F  A  N  Q  A  G

                 2314      2324      2334      2344      2354      2364      2374      2384      2394
2305     CTCGACATGCTGGAGACGACGTTGATTGCTCTCCAGGACATCTCCCTTGAGAAGATCCTTGATGATGATGGCAGGAAGGCGCTCTGCACTGAGTAC
769       L  D  M  L  E  T  T  L  I  A  L  Q  D  I  S  L  E  K  I  L  D  D  D  G  R  K  A  L  C  T  E  Y

                 2410      2420      2430      2440      2450      2460      2470      2480      2490
2401     CCTAAGATTATGCAGCAGGGCTTCGCATACCTCCCCGGCGGCGTGTGTGTATCGAGCATGGGGCGGCCGGTGTCGTACGAGCAGGCGGTGGCGTGG
801       P  K  I  M  Q  Q  G  F  A  Y  L  P  G  G  V  C  V  S  S  M  G  R  P  V  S  Y  E  Q  A  V  A  W

                 2506      2516      2526      2536      2546      2556
2497     AAGGTCCTGAGCGACGACGACACCCCGCACTGCCTCGCCTTCATGTTCGTGAACTGGTCCTTCGTGTGA
833       K  V  L  S  D  D  D  T  P  H  C  L  A  F  M  F  V  N  W  S  F  V  *
```

Supplementary Figure S6 ORF sequence of *SsHB15* and its encoding protein. The protein marked with yellow color represents the Homeodomain (PF00046)
