# Supplementary material for: Expression Profiling and MicroRNA Regulatory Networks of Homeobox Family Genes in Sugarcane Saccharum spontaneum L
Source: Int J Mol Sci. 2022 Aug 5;23(15):8724. doi: 10.3390/ijms23158724 (PMC9369071; doi:10.3390/ijms23158724)
Supplement: Supplementary file 1 [file ijms-23-08724-s001.zip › Supplementary file 15.pdf]

```
                   10        20        30        40        50        60        70        80        90
1          ATGGCGTTCAACTACCACGACCACGTGCTGGCGATGGACCCCGCCGCGGTTGGGGCCGCGGCCGCGAACCCTAGCTTTGTCCCCGGAGGTGGTGGT
1           M  A  F  N  Y  H  D  H  V  L  A  M  D  P  A  A  V  G  A  A  A  A  N  P  S  F  V  P  G  G  G  G

                  106       116       126       136       146       156       166       176       186
97         GGCGTGGCCGGTGGGGGGCCGGGCTGGGAGAGGGAGAAGGCGGCCATCGCGGCGCACCCGCTGTACGAGCGCCTGCTCGAGGCGCACGTCGCCTGC
33          G  V  A  G  G  G  P  G  W  E  R  E  K  A  A  I  A  A  H  P  L  Y  E  R  L  L  E  A  H  V  A  C

                  202       212       222       232       242       252       262       272       282
193        CTCCGCGTCGCCACCCCCGTGGACCAGCTCCCGCGCATCGACGCCCAGATAGCCGCGAGGCCCCCGCCCATGGCGGCCGCCGCCGCGGCTGCGGCG
65          L  R  V  A  T  P  V  D  Q  L  P  R  I  D  A  Q  I  A  A  R  P  P  P  M  A  A  A  A  A  A  A  A

                  298       308       318       328       338       348       358       368       378
289        GCGGGCGGAGCACACTCGGGAGGCGAGGAGCTCGACCTCTTCATGACGCATTATGTGCTGCTCCTCTGTTCATTCAAGGAACAACTCCAGCAGCAC
97          A  G  G  A  H  S  G  G  E  E  L  D  L  F  M  T  H  Y  V  L  L  L  C  S  F  K  E  Q  L  Q  Q  H

                  394       404       414       424       434       444       454       464       474
385        GTGCGTGTTCATGCGATGGAAGCAGTCATGGCTTGCTGGGAGCTTGAACAAACTTTACAAAGCCTTACAGGGGCATCTCCTGGTGAGGGCACTGGG
129         V  R  V  H  A  M  E  A  V  M  A  C  W  E  L  E  Q  T  L  Q  S  L  T  G  A  S  P  G  E  G  T  G

                  490       500       510       520       530       540       550       560       570
481        GCAACTATGTCTGATGATGAAGACAATCAGGTAGACAGTGAGAGCAACATGTTTGATGGAAATGAGGGGTCAGATGGAATGGGATTTGGTCCATTA
161         A  T  M  S  D  D  E  D  N  Q  V  D  S  E  S  N  M  F  D  G  N  E  G  S  D  G  M  G  F  G  P  L

                  586       596       606       616       626       636       646       656       666
577        ATGTTGACTGAGGGTGAAAGATCCTTAGTTGAGCGTGTCCGGCAGGAGCTAAAGCATGAGCTTAAGCAGGGGTATAGAGACAAACTTGTGGATATT
193         M  L  T  E  G  E  R  S  L  V  E  R  V  R  Q  E  L  K  H  E  L  K  Q  G  Y  R  D  K  L  V  D  I

                  682       692       702       712       722       732       742       752       762
673        CGGGAAGAGATAATGCGGAAGCGAAGAGCTGGGAAACTCCCTGGCGACACTGCTTCTACCTTAAAAGCTTGGTGGCAAGCTCATTCTAAGTGGCCA
225         R  E  E  I  M  R  K  R  R  A  G  K  L  P  G  D  T  A  S  T  L  K  A  W  W  Q  A  H  S  K  W  P

                  778       788       798       808       818       828       838       848       858
769        TACCCAACCGAGGAAGACAAGGCTCGACTGGTGCAGGAAACAGGGCTACAACTGAAGCAGATCAATAACTGGTTTATCAACCAGCGTAAGCGGAAC
257         Y  P  T  E  E  D  K  A  R  L  V  Q  E  T  G  L  Q  L  K  Q  I  N  N  W  F  I  N  Q  R  K  R  N

                  874       884       894       904       914       924       934
865        TGGCACAACAATCCGGCGTCATCCTCATCGGACAAAAGCAAGAGAAAAAGGTACGGTCACAAGAAACTAGACTAA
289         W  H  N  N  P  A  S  S  S  S  D  K  S  K  R  K  R  Y  G  H  K  K  L  D  *
```

Supplementary Figure S7 ORF sequence of *SsHB97* and its encoding protein. The protein marked with yellow color represents the Homeobox_KN (PF05920)
