# Supplementary material for: Expression Profiling and MicroRNA Regulatory Networks of Homeobox Family Genes in Sugarcane Saccharum spontaneum L
Source: Int J Mol Sci. 2022 Aug 5;23(15):8724. doi: 10.3390/ijms23158724 (PMC9369071; doi:10.3390/ijms23158724)
Supplement: Supplementary file 1 [file ijms-23-08724-s001.zip › Supplementary file 16.pdf]

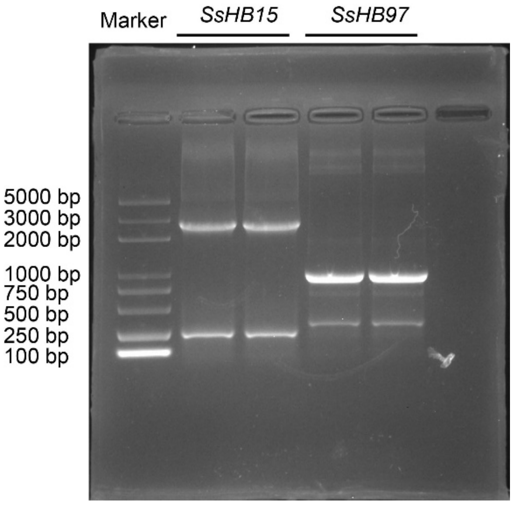

Supplementary Figure S8 The original image of 1% agarose gel electrophoresis of PCR clones of *SsHB15* and *SsHB97* coding sequences
