# Supplementary material for: Expression Profiling and MicroRNA Regulatory Networks of Homeobox Family Genes in Sugarcane Saccharum spontaneum L
Source: Int J Mol Sci. 2022 Aug 5;23(15):8724. doi: 10.3390/ijms23158724 (PMC9369071; doi:10.3390/ijms23158724)
Supplement: Supplementary file 1 [file ijms-23-08724-s001.zip › Supplementary file 5.pdf]

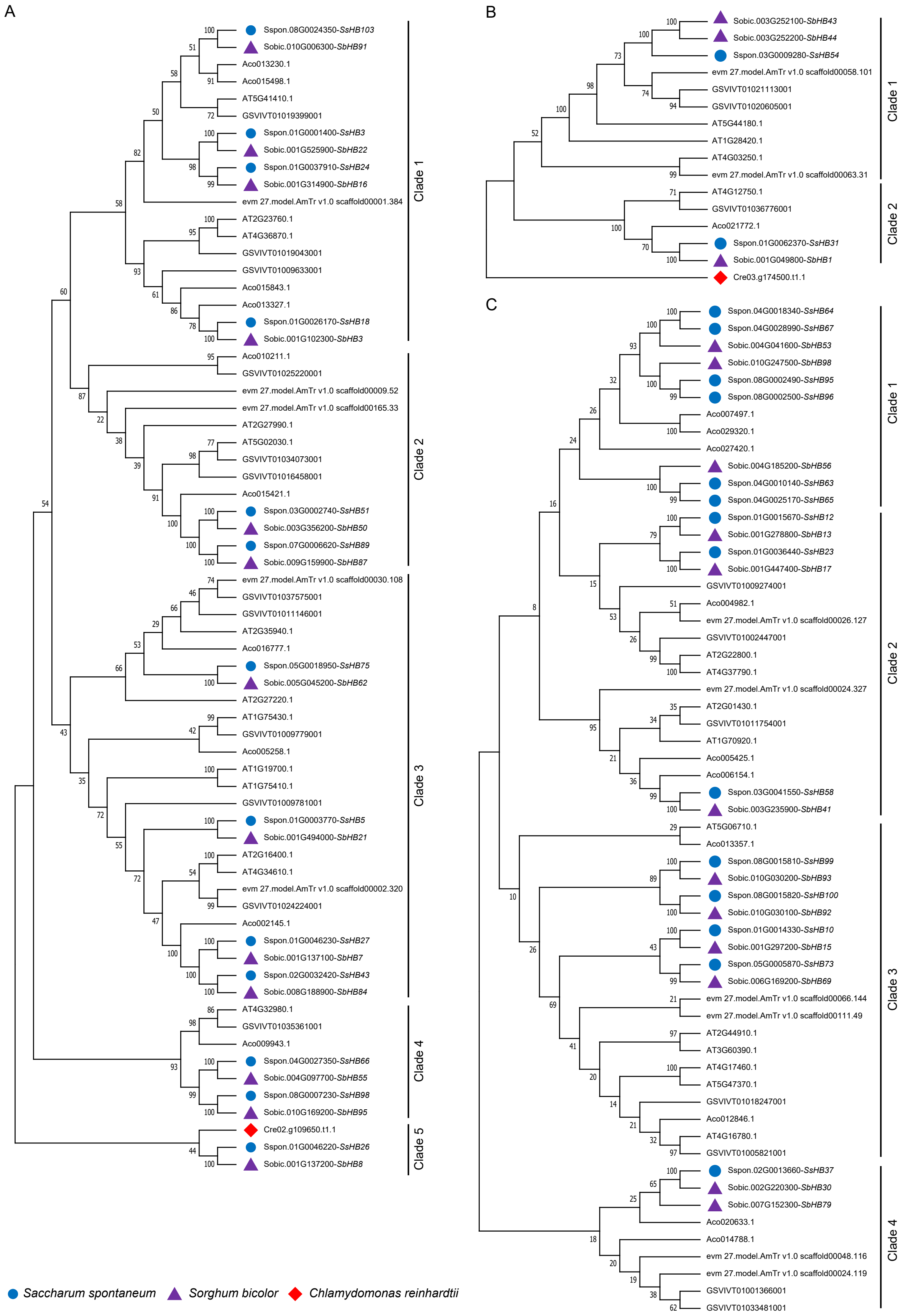

A
Sspon.08G0024350-SsHB103
Sobic.010G006300-SbHB91
Aco013230.1
Aco015498.1
AT5G41410.1
GSVIVT01019399001
Sspon.01G0001400-SsHB3
Sobic.001G525900-SbHB22
Sspon.01G0037910-SsHB24
Sobic.001G314900-SbHB16
evm 27.model.AmTr v1.0 scaffold00001.384
AT2G23760.1
AT4G36870.1
GSVIVT01019043001
GSVIVT01009633001
Aco015843.1
Aco013327.1
Sspon.01G0026170-SsHB18
Sobic.001G102300-SbHB3
Aco010211.1
GSVIVT01025220001
evm 27.model.AmTr v1.0 scaffold00009.52
evm 27.model.AmTr v1.0 scaffold00165.33
AT2G27990.1
AT5G02030.1
GSVIVT01034073001
GSVIVT01016458001
Aco015421.1
Sspon.03G0002740-SsHB51
Sobic.003G356200-SbHB50
Sspon.07G0006620-SsHB89
Sobic.009G159900-SbHB87
evm 27.model.AmTr v1.0 scaffold00030.108
GSVIVT01037575001
GSVIVT01011146001
AT2G35940.1
Aco016777.1
Sspon.05G0018950-SsHB75
Sobic.005G045200-SbHB62
AT2G27220.1
AT1G75430.1
GSVIVT01009779001
Aco005258.1
AT1G19700.1
AT1G75410.1
GSVIVT01009781001
Sspon.01G0003770-SsHB5
Sobic.001G494000-SbHB21
AT2G16400.1
AT4G34610.1
evm 27.model.AmTr v1.0 scaffold00002.320
GSVIVT01024224001
Aco002145.1
Sspon.01G0046230-SsHB27
Sobic.001G137100-SbHB7
Sspon.02G0032420-SsHB43
Sobic.008G188900-SbHB84
AT4G32980.1
GSVIVT01035361001
Aco009943.1
Sspon.04G0027350-SsHB66
Sobic.004G097700-SbHB55
Sspon.08G0007230-SsHB98
Sobic.010G169200-SbHB95
Cre02.g109650.t1.1
Sspon.01G0046220-SsHB26
Sobic.001G137200-SbHB8
Clade 1
Clade 2
Clade 3
Clade 4
Clade 5
B
Sobic.003G252100-SbHB43
Sobic.003G252200-SbHB44
Sspon.03G0009280-SsHB54
evm 27.model.AmTr v1.0 scaffold00058.101
GSVIVT01021113001
GSVIVT01020605001
AT5G44180.1
AT1G28420.1
AT4G03250.1
evm 27.model.AmTr v1.0 scaffold00063.31
AT4G12750.1
GSVIVT01036776001
Aco021772.1
Sspon.01G0062370-SsHB31
Sobic.001G049800-SbHB1
Cre03.g174500.t1.1
Clade 1
Clade 2
C
Sspon.04G0018340-SsHB64
Sspon.04G0028990-SsHB67
Sobic.004G041600-SbHB53
Sobic.010G247500-SbHB98
Sspon.08G0002490-SsHB95
Sspon.08G0002500-SsHB96
Aco007497.1
Aco029320.1
Aco027420.1
Sobic.004G185200-SbHB56
Sspon.04G0010140-SsHB63
Sspon.04G0025170-SsHB65
Sspon.01G0015670-SsHB12
Sobic.001G278800-SbHB13
Sspon.01G0036440-SsHB23
Sobic.001G447400-SbHB17
GSVIVT01009274001
Aco004982.1
evm 27.model.AmTr v1.0 scaffold00026.127
GSVIVT01002447001
AT2G22800.1
AT4G37790.1
evm 27.model.AmTr v1.0 scaffold00024.327
AT2G01430.1
GSVIVT01011754001
AT1G70920.1
Aco005425.1
Aco006154.1
Sspon.03G0041550-SsHB58
Sobic.003G235900-SbHB41
AT5G06710.1
Aco013357.1
Sspon.08G0015810-SsHB99
Sobic.010G030200-SbHB93
Sspon.08G0015820-SsHB100
Sobic.010G030100-SbHB92
Sspon.01G0014330-SsHB10
Sobic.001G297200-SbHB15
Sspon.05G0005870-SsHB73
Sobic.006G169200-SbHB69
evm 27.model.AmTr v1.0 scaffold00066.144
evm 27.model.AmTr v1.0 scaffold00111.49
AT2G44910.1
AT3G60390.1
AT4G17460.1
AT5G47370.1
GSVIVT01018247001
Aco012846.1
AT4G16780.1
GSVIVT01005821001
Sspon.02G0013660-SsHB37
Sobic.002G220300-SbHB30
Sobic.007G152300-SbHB79
Aco020633.1
Aco014788.1
evm 27.model.AmTr v1.0 scaffold00048.116
evm 27.model.AmTr v1.0 scaffold00024.119
GSVIVT01001366001
GSVIVT01033481001
Clade 1
Clade 2
Clade 3
Clade 4
Saccharum spontaneum
Sorghum bicolor
Chlamydomonas reinhardtii

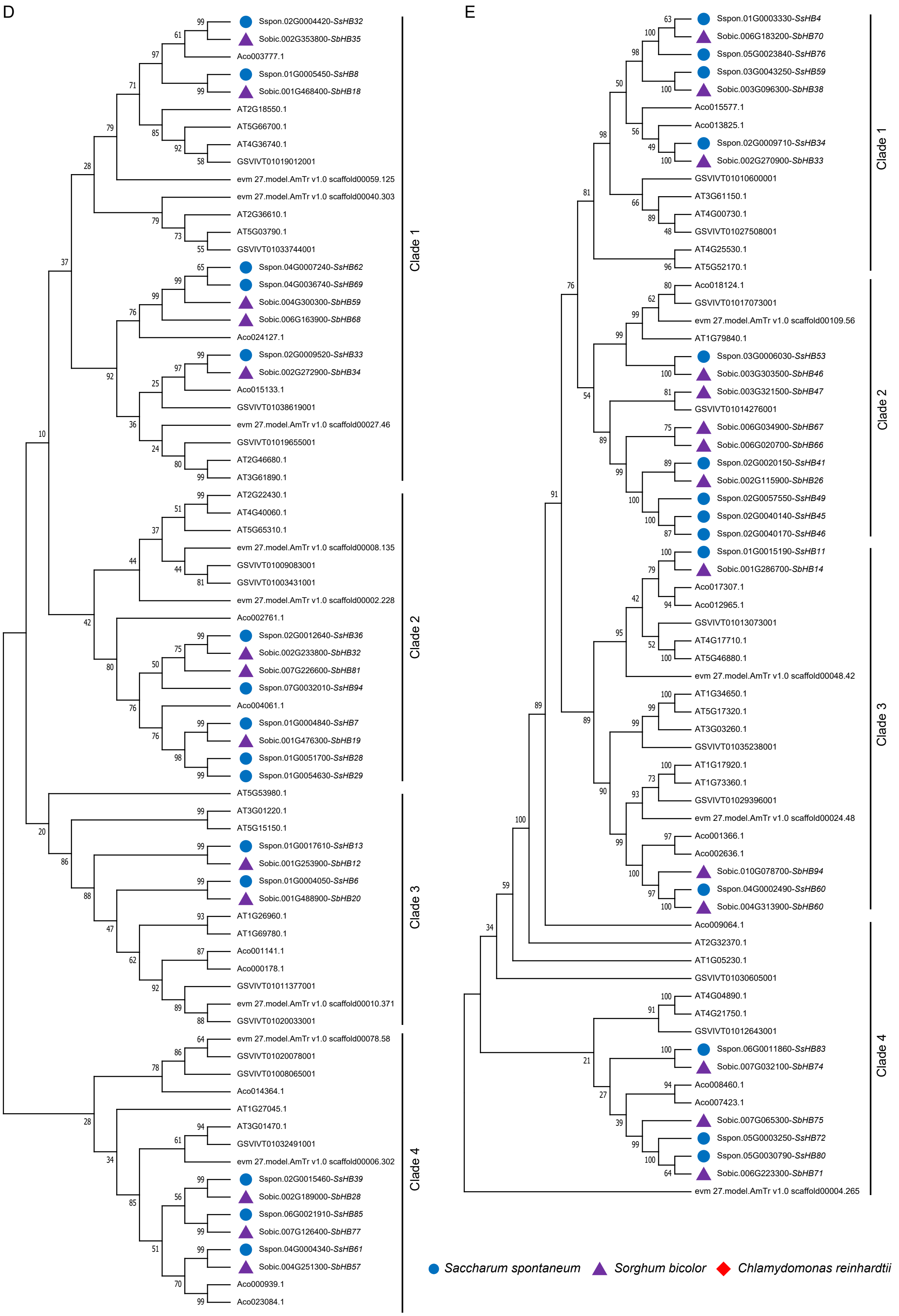

D
Sspon.02G0004420-SsHB32
Sobic.002G353800-SbHB35
Aco003777.1
Sspon.01G0005450-SsHB8
Sobic.001G468400-SbHB18
AT2G18550.1
AT5G66700.1
AT4G36740.1
GSVIVT01019012001
evm 27.model.AmTr v1.0 scaffold00059.125
evm 27.model.AmTr v1.0 scaffold00040.303
AT2G36610.1
AT5G03790.1
GSVIVT01033744001
Sspon.04G0007240-SsHB62
Sspon.04G0036740-SsHB69
Sobic.004G300300-SbHB59
Sobic.006G163900-SbHB68
Aco024127.1
Sspon.02G0009520-SsHB33
Sobic.002G272900-SbHB34
Aco015133.1
GSVIVT01038619001
evm 27.model.AmTr v1.0 scaffold00027.46
GSVIVT01019655001
AT2G46680.1
AT3G61890.1
Clade 1
AT2G22430.1
AT4G40060.1
AT5G65310.1
evm 27.model.AmTr v1.0 scaffold00008.135
GSVIVT01009083001
GSVIVT01003431001
evm 27.model.AmTr v1.0 scaffold00002.228
Aco002761.1
Sspon.02G0012640-SsHB36
Sobic.002G233800-SbHB32
Sobic.007G226600-SbHB81
Sspon.07G0032010-SsHB94
Aco004061.1
Sspon.01G0004840-SsHB7
Sobic.001G476300-SbHB19
Sspon.01G0051700-SsHB28
Sspon.01G0054630-SsHB29
Clade 2
AT5G53980.1
AT3G01220.1
AT5G15150.1
Sspon.01G0017610-SsHB13
Sobic.001G253900-SbHB12
Sspon.01G0004050-SsHB6
Sobic.001G488900-SbHB20
AT1G26960.1
AT1G69780.1
Aco001141.1
Aco000178.1
GSVIVT01011377001
evm 27.model.AmTr v1.0 scaffold00010.371
GSVIVT01020033001
Clade 3
evm 27.model.AmTr v1.0 scaffold00078.58
GSVIVT01020078001
GSVIVT01008065001
Aco014364.1
AT1G27045.1
AT3G01470.1
GSVIVT01032491001
evm 27.model.AmTr v1.0 scaffold00006.302
Sspon.02G0015460-SsHB39
Sobic.002G189000-SbHB28
Sspon.06G0021910-SsHB85
Sobic.007G126400-SbHB77
Sspon.04G0004340-SsHB61
Sobic.004G251300-SbHB57
Aco000939.1
Aco023084.1
Clade 4
E
Sspon.01G0003330-SsHB4
Sobic.006G183200-SbHB70
Sspon.05G0023840-SsHB76
Sspon.03G0043250-SsHB59
Sobic.003G096300-SbHB38
Aco015577.1
Aco013825.1
Sspon.02G0009710-SsHB34
Sobic.002G270900-SbHB33
GSVIVT01010600001
AT3G61150.1
AT4G00730.1
GSVIVT01027508001
AT4G25530.1
AT5G52170.1
Clade 1
Aco018124.1
GSVIVT01017073001
evm 27.model.AmTr v1.0 scaffold00109.56
AT1G79840.1
Sspon.03G0006030-SsHB53
Sobic.003G303500-SbHB46
Sobic.003G321500-SbHB47
GSVIVT01014276001
Sobic.006G034900-SbHB67
Sobic.006G020700-SbHB66
Sspon.02G0020150-SsHB41
Sobic.002G115900-SbHB26
Sspon.02G0057550-SsHB49
Sspon.02G0040140-SsHB45
Sspon.02G0040170-SsHB46
Clade 2
Sspon.01G0015190-SsHB11
Sobic.001G286700-SbHB14
Aco017307.1
Aco012965.1
GSVIVT01013073001
AT4G17710.1
AT5G46880.1
evm 27.model.AmTr v1.0 scaffold00048.42
AT1G34650.1
AT5G17320.1
AT3G03260.1
GSVIVT01035238001
AT1G17920.1
AT1G73360.1
GSVIVT01029396001
evm 27.model.AmTr v1.0 scaffold00024.48
Aco001366.1
Aco002636.1
Sobic.010G078700-SbHB94
Sspon.04G0002490-SsHB60
Sobic.004G313900-SbHB60
Clade 3
Aco009064.1
AT2G32370.1
AT1G05230.1
GSVIVT01030605001
AT4G04890.1
AT4G21750.1
GSVIVT01012643001
Sspon.06G0011860-SsHB83
Sobic.007G032100-SbHB74
Aco008460.1
Aco007423.1
Sobic.007G065300-SbHB75
Sspon.05G0003250-SsHB72
Sspon.05G0030790-SsHB80
Sobic.006G223300-SbHB71
evm 27.model.AmTr v1.0 scaffold00004.265
Clade 4
Saccharum spontaneum
Sorghum bicolor
Chlamydomonas reinhardtii

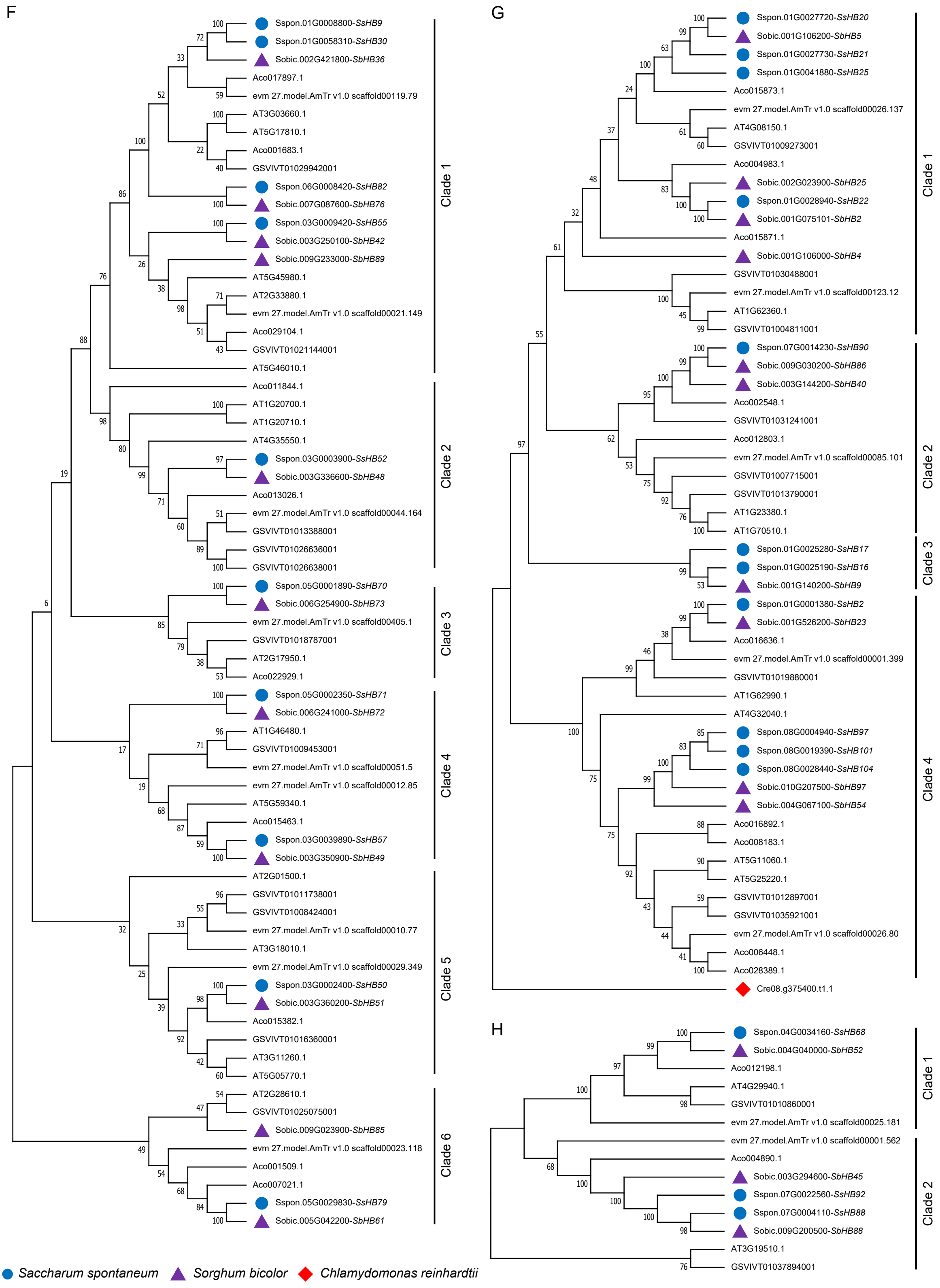

F
Sspon.01G0008800-SsHB9
Sspon.01G0058310-SsHB30
Sobic.002G421800-SbHB36
Aco017897.1
evm 27.model.AmTr v1.0 scaffold00119.79
AT3G03660.1
AT5G17810.1
Aco001683.1
GSVIVT01029942001
Sspon.06G0008420-SsHB82
Sobic.007G087600-SbHB76
Sspon.03G0009420-SsHB55
Sobic.003G250100-SbHB42
Sobic.009G233000-SbHB89
AT5G45980.1
AT2G33880.1
evm 27.model.AmTr v1.0 scaffold00021.149
Aco029104.1
GSVIVT01021144001
AT5G46010.1
Clade 1
Aco011844.1
AT1G20700.1
AT1G20710.1
AT4G35550.1
Sspon.03G0003900-SsHB52
Sobic.003G336600-SbHB48
Aco013026.1
evm 27.model.AmTr v1.0 scaffold00044.164
GSVIVT01013388001
GSVIVT01026636001
GSVIVT01026638001
Clade 2
Sspon.05G0001890-SsHB70
Sobic.006G254900-SbHB73
evm 27.model.AmTr v1.0 scaffold00405.1
GSVIVT01018787001
AT2G17950.1
Aco022929.1
Clade 3
Sspon.05G0002350-SsHB71
Sobic.006G241000-SbHB72
AT1G46480.1
GSVIVT01009453001
evm 27.model.AmTr v1.0 scaffold00051.5
evm 27.model.AmTr v1.0 scaffold00012.85
AT5G59340.1
Aco015463.1
Sspon.03G0039890-SsHB57
Sobic.003G350900-SbHB49
Clade 4
AT2G01500.1
GSVIVT01011738001
GSVIVT01008424001
evm 27.model.AmTr v1.0 scaffold00010.77
AT3G18010.1
evm 27.model.AmTr v1.0 scaffold00029.349
Sspon.03G0002400-SsHB50
Sobic.003G360200-SbHB51
Aco015382.1
GSVIVT01016360001
AT3G11260.1
AT5G05770.1
Clade 5
AT2G28610.1
GSVIVT01025075001
Sobic.009G023900-SbHB85
evm 27.model.AmTr v1.0 scaffold00023.118
Aco001509.1
Aco007021.1
Sspon.05G0029830-SsHB79
Sobic.005G042200-SbHB61
Clade 6
G
Sspon.01G0027720-SsHB20
Sobic.001G106200-SbHB5
Sspon.01G0027730-SsHB21
Sspon.01G0041880-SsHB25
Aco015873.1
evm 27.model.AmTr v1.0 scaffold00026.137
AT4G08150.1
GSVIVT01009273001
Aco004983.1
Sobic.002G023900-SbHB25
Sspon.01G0028940-SsHB22
Sobic.001G075101-SbHB2
Aco015871.1
Sobic.001G106000-SbHB4
GSVIVT01030488001
evm 27.model.AmTr v1.0 scaffold00123.12
AT1G62360.1
GSVIVT01004811001
Clade 1
Sspon.07G0014230-SsHB90
Sobic.009G030200-SbHB86
Sobic.003G144200-SbHB40
Aco002548.1
GSVIVT01031241001
Aco012803.1
evm 27.model.AmTr v1.0 scaffold00085.101
GSVIVT01007715001
GSVIVT01013790001
AT1G23380.1
AT1G70510.1
Clade 2
Sspon.01G0025280-SsHB17
Sspon.01G0025190-SsHB16
Sobic.001G140200-SbHB9
Clade 3
Sspon.01G0001380-SsHB2
Sobic.001G526200-SbHB23
Aco016636.1
evm 27.model.AmTr v1.0 scaffold00001.399
GSVIVT01019880001
AT1G62990.1
AT4G32040.1
Sspon.08G0004940-SsHB97
Sspon.08G0019390-SsHB101
Sspon.08G0028440-SsHB104
Sobic.010G207500-SbHB97
Sobic.004G067100-SbHB54
Aco016892.1
Aco008183.1
AT5G11060.1
AT5G25220.1
GSVIVT01012897001
GSVIVT01035921001
evm 27.model.AmTr v1.0 scaffold00026.80
Aco006448.1
Aco028389.1
Clade 4
Cre08.g375400.t1.1
H
Sspon.04G0034160-SsHB68
Sobic.004G040000-SbHB52
Aco012198.1
AT4G29940.1
GSVIVT01010860001
evm 27.model.AmTr v1.0 scaffold00025.181
Clade 1
evm 27.model.AmTr v1.0 scaffold00001.562
Aco004890.1
Sobic.003G294600-SbHB45
Sspon.07G0022560-SsHB92
Sspon.07G0004110-SsHB88
Sobic.009G200500-SbHB88
AT3G19510.1
GSVIVT01037894001
Clade 2
Saccharum spontaneum
Sorghum bicolor
Chlamydomonas reinhardtii

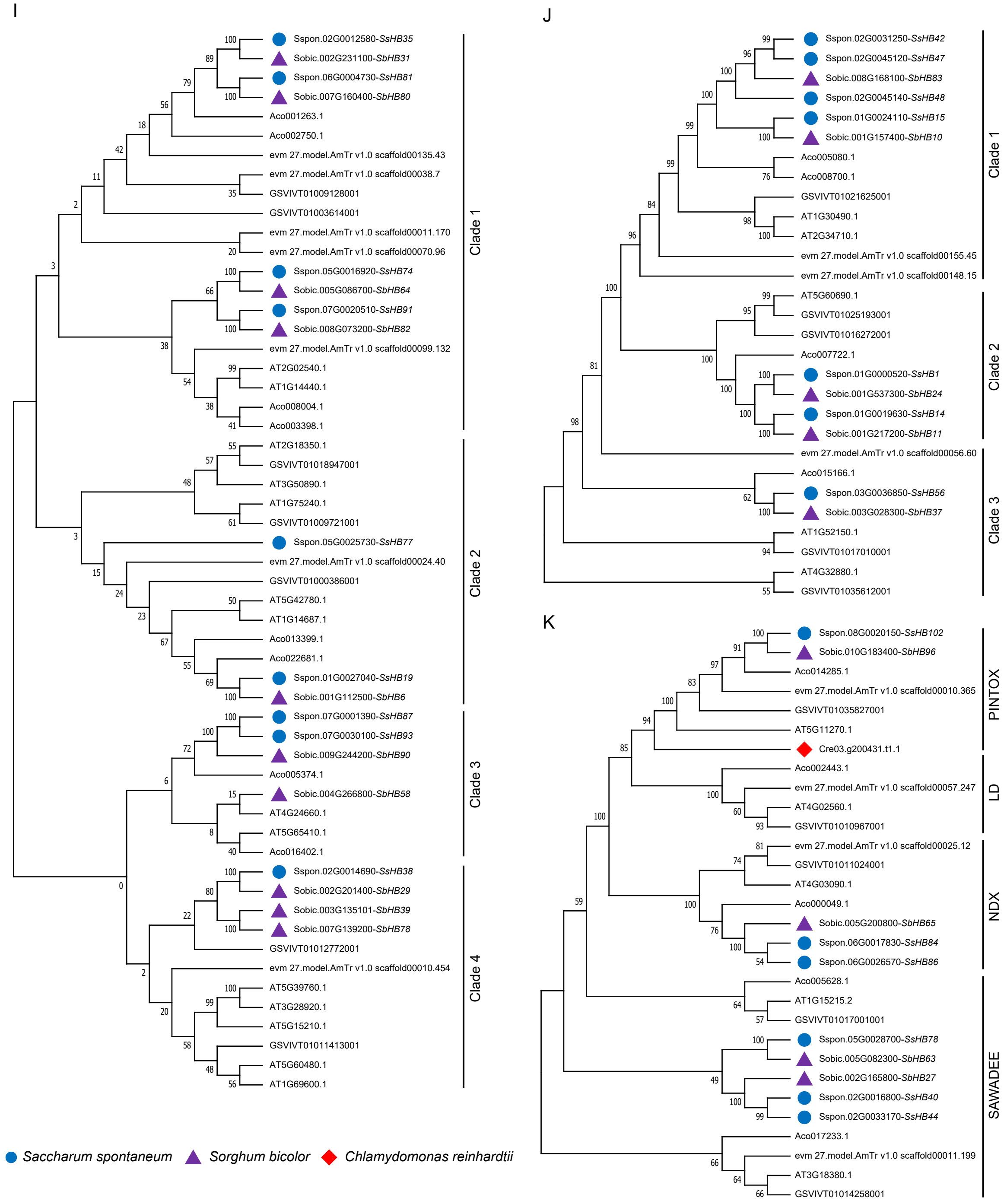

Supplementary Figure S2 Phylogenetic tree of HB subfamilies among representative monocotyledons and dicotyledons. The phylogenetic trees were built with the NJ algorithm for BEL (A), DDT (B), HD-ZIP II (C), HD-ZIP I (D), HD-ZIP IV (E), WOX (F), KNOX (G), PHD (H), PLINC (I), HD-ZIP III (J), LD, SAWADEE, NDX and PINTOX (K)
