# Supplementary material for: Expression Profiling and MicroRNA Regulatory Networks of Homeobox Family Genes in Sugarcane Saccharum spontaneum L
Source: Int J Mol Sci. 2022 Aug 5;23(15):8724. doi: 10.3390/ijms23158724 (PMC9369071; doi:10.3390/ijms23158724)
Supplement: Supplementary file 1 [file ijms-23-08724-s001.zip › Supplementary file 6.pdf]

```
SsHB3-1   : --------WRPQRGLPERAVTILRAWLFEHFLH-PYPSDVDKHILARQT----------GLSRSQVSNWFINARVRL-WKPMVEE-   BEL
SsHB24    : --------WRPQRGLPERAVSILRAWLFEHFLH-PYPSDVDKHILARQT----------GLSRNQVSNWFINARVRL-WKPMVEE-
SsHB51-1  : --------WRPQRGLPERAVSVLRAWLFEHFLH-PYPTDGDKQMLAKQT----------GLTRNQVSNWFINARVRL-WKPMVEE-
SsHB75-2  : --------WRPQRGLPERSVSILRAWLFEHFLH-PYPKDSDKIMLAKQT----------GLTRSQVSNWFINARVRL-WKPMVEE-
SsHB89-1  : --------WRPQRGLPERAVSVLRSWLFEHFLH-PYPTDSDKQMLAKQT----------GLTRNQVSNWFINARVRL-WKPMVEE-
SbHB50    : --------WRPQRGLPERAVSVLRAWLFEHFLH-PYPTDGDKQMLAKQT----------GLTRNQVSNWFINARVRL-WKPMVEE-
AT2G35940 : --------WRPQRGLPERAVSVLRAWLFEHFLH-PYPKDSDKHMLAKQT----------GLTRSQVSNWFINARVRL-WKPMVEE-
SsHB31-1  : -------RDNAGAKKSPQQIQMLEKFYSDV----QYPKPEEMGEYATCV----------GLTYSQVRIWFKERRRKDRRREMGVI-   DDT
SsHB54-1  : -------KPVKRMMKTPYQLEVLEKTYAVE----QYPSEAMRLELSAKI----------GLSDRQLQMWFCHRRLKD-RKP-----
SbHB1     : -------RDNAGAKKSPQQIQMLEKFYSDA----QYPKPEEMGEYATCV----------GLTYSQVRIWFKERRRKE-RREMGAI-
AT4G03250 : -------NSKKRKLKTPMQVMALENFYNEH----KYPTEEMKGKLAEEV----------GLTEKQVSGWFCHRRLKD-KRHVKED-
SsHB6     : --------GEKKRRLNVEQVRTLEKNFELG----NKLEPERKLQLARAL----------GLQPRQVAIWFQNRRARW-KTKQLEK-   HD-ZIP I
SsHB13-1  : --------GERKRRLSVEQVRTLERSFEVA----NKLEPERKAQLARAL----------GLQPRQVAIWFQNRRARW-KTKQLEK-
SsHB36-1  : --------GEKKRRLSAEQVRALERSFEVE----NKLEPERKARLARDL----------GLQPRQVAVWFQNRRARW-KTKQLER-
SsHB61-1  : --------PEKKRRLTPEQVHLLERSFEEE----NKLEPERKTELARKL----------GLQPRQVAVWFQNRRARW-KTKQLER-
SsHB85-1  : --------PEKKRRLTAEQVQLLERSFEEE----NKLEPERKTELARRL----------GMAPRQVAVWFQNRRARW-KTKQLET-
SbHB77    : --------PEKKRRLTAEQVQLLERSFEEE----NKLEPERKTELARRL----------GMAPRQVAVWFQNRRARW-KTKQLET-
AT3G01470 : --------PEKKRRLTTEQVHLLEKSFETE----NKLEPERKTQLAKKL----------GLQPRQVAVWFQNRRARW-KTKQLER-
SsHB10    : --------SRKKLRLSKDQAAVLEDSFKEH----NTLNPKQKAALAKQL----------NLKPRQVEVWFQNRRART-KLKQTEV-   HD-ZIP II
SsHB12-1  : --------SRKKLRLSKEQSALLEDHFKEH----STLNPKQKAALARQL----------NLRPRQVEVWFQNRRART-KLKQTEV-
SsHB23-1  : --------TRKKLRLTKEQSALLEDRFKEH----STLNPKQKVALAKQL----------NLRPRQVEVWFQNRRART-KLKQTEV-
SsHB73-1  : --------SRKKLRLSKDQSAVLEDSFREH----PTLNPRQKAALAQQL----------GLRPRQVEVWFQNRRART-KLKQTEV-
SsHB99-1  : --------GRKKLRLSKEQAAVLEECFKTH----STLNPKQKLALATRL----------GLRPRQVEVWFQNRRART-KLKQTEV-
SbHB17    : --------TRKKLRLTKEQSALLEDRFKEH----STLNPKQKVALAKQL----------NLRPRQVEVWFQNRRART-KLKQTEV-
AT5G06710 : --------TRKKLRLSKDQSAFLEDSFKEH----STLNPKQKIALAKQL----------NLRPRQVEVWFQNRRART-KLKQTEV-
SsHB1-1   : -------DSGKYVRYTPEQVEVLERLYIDC----PKPTSSRRQQLLRECPI------LSNIEPKQIKVWFQNRRCRD-KQRKESSR   HD-ZIP III
SsHB14-2  : -------DTGKYVRYTPEQVEALERVYAEC----PKPSSARRQQLLRECPI------LSNIEPKQIKVWFQNRRCRD-KQRNESSR
SsHB15-1  : -------DTGKYVRYTPEQVEALERVYSEC----PKPSSLRRQQLIRECPI------LSNIEPKQIKVWFQNRRCRE-KQRKEASR
SsHB42-1  : -------DAGKYVRYTPEQVEALERVYSEC----PKPSSLRRQQLIRDCPI------LSNIEPKQIKVWFQNRRCRE-KQRKESSR
SsHB48    : -------DAGKYVRYTPEQVEALERVYSEC----PKPSSLRRQQLIRDCPI------LSNIEPKQIKVWFQNRRCRE-KQRKESSR
SbHB24    : -------DSGKYVRYTPEQVEVLERLYIDC----PKPSSSRRQQLLRECPI------LSNIEPKQIKVWFQNRRCRD-KQRKESSR
AT1G52150 : -------DNGKYVRYTPEQVEALERLYHDC----PKPSSIRRQQLIRECPI------LSNIEPKQIKVWFQNRRCRE-KQRKEASR
SsHB11-1  : G------KKKRYHRHTAHQIQQMEALFKEC----PHPDDKQRLKLSQEL----------GLKPRQVKFWFQNRRTQM-KAQQDRA-   HD-ZIP IV
SsHB59-1  : K------RKKRYHRHTPHQIQELEALFKEC----PHPDEKQRGELSKRL----------GLDPRQVKFWFQNRRTQM-KTQLERH-
SsHB60-4  : R------RKKRYHRHTPRQIQQLEAMFKEC----PHPDENQRMHLSREL----------GLEPRQIKFWFQNRRTQM-KAQHERQ-
SsHB80-1  : S------KKKRYHRHTQHQIQEMEAFFKEC----PHPDDKQRKELSREL----------GLEPLQVKFWFQNKRTQM-KNQHERQ-
SsHB83-1  : A------RKKRYHRHTQHQIQELEAFFKEY----PHPDDKQRKELSREL----------GLEPLQVKFWFQNKRTQM-KTQQERH-
SbHB74    : A------RKKRYHRHTQHQIQELEAFFKEY----PHPDDKQRKELSREL----------GLEPLQVKFWFQNKRTQM-KTQQERH-
AT1G05230 : N------KKKRYHRHTQLQIQEMEAFFKEC----PHPDDKQRKQLSREL----------NLEPLQVKFWFQNKRTQM-KNHHERH-
SsHB2-1   : -------RKRRAGKLPGDTTSILKQWWQQHSKW-PYPTEDDKAKLVEET----------GLQLKQINNWFINQRKRN-WHNNSQT-   KNOX
SsHB90-1  : -------KKRKKGKLPKDARSALMEWWNTHYRW-PYPTEEDKVRLAAMT----------GLDPKQINNWFINQRKRH-WKPSEDM-
SsHB97-1  : -------RKRRAGKLPGDTASTLKAWWQAHSKW-PYPTEEDKARLVQET----------GLQLKQINNWFINQRKRN-WHNNPAS-
SsHB101-1 : -------RKRRAGKLPGDTASTLKAWWQAHSKW-PYPTEEDKARLVQET----------GLQLKQINNWFINQRKRN-WHNNPAS-
SsHB104   : -------RKRRAGKLPGDTASTLKAWWQAHSKW-PYPTEEDKARLVQET----------GLQLKQINNWFINQRKRN-WHNNPAS-
SbHB97    : -------RKRRAGKLPGDTASTLKAWWQAHSKW-PYPTEEDKARLVQET----------GLQLKQINNWFINQRKRN-WHNNPAS-
AT1G62990 : -------RKRRAGKLPGDTTTVLKNWWQQHCKW-PYPTEDDKAKLVEET----------GLQLKQINNWFINQRKRN-WHNNSHS-
SsHB84-3  : -------KRRKRTLMNNEQIDELEKALVDEPE--MHKNAVLLQSWSEKLSL----QGS-EITASQLKNWLNNRKAKL-AR------   NDX
SsHB86-1  : -------KRRKRTLMNNEQIDELEKALVDEPE--MHKNAVLLQSWSEKLSL----QGS-EITASQLKNWLNNRKAKL-AR------
SbHB65    : -------KRRKRTLMNNEQIDELEKALVDEPE--MHKNAVLLQSWSEKLSL----QGS-EITASQLKNWLNNRKAKL-AR------
AT4G03090 : -------KKRKRSIMNADQMGMIEKALAEEPD--LQRNSASRQLWADKISQ----KGSEVITSSQLKNWLNNRKAKL-AR------
SsHB68-1  : ----------KLFRIPPAAVEVLRKAFAEN----ELPPRDVKENLSREL----------GISFEKIDKWFKNTRCAA-LRDRKAE-   PHD
SsHB88-1  : ----------RKGHFGPVVNQKLYEHFKTQ----QYPSRSVKERLAEEL----------GLTFRQVSRWFETRRHFA---------
SsHB92-4  : ----------RKGHFGPVVNQKLYEHFKTQ----QYPSRSVKESLAEEL----------GLTFRQVSKWFETRRHFA---------
SbHB52    : ----------KLFRIPPAAVEVLRRAFAEN----ELPPRDVKENLSREL----------GISFEKIDKWFKNTRCAA-LRDRKAE-
AT4G29940 : ----------RMFRLPRNAVEKLRQVFAET----ELPSKAVRDRLAKEL----------SLDPEKVNKWFKNTRYMA-LRNRKTE-
SsHB102-2 : -------EWSARKRLKKVQLETLERVYLRS----KRPTNTMISSIVQVT----------NLPRKTIVKWFEDRREQD-GVPDHRV-   PINTOX
SbHB96    : -------EWSARKRLKKVQLETLERVYLRS----KRPTNAMISSIVQVT----------NLPRKTIVKWFEDRREQD-GVPDHRV-
AT5G11270 : -------RWSAQKRVKKAHIETLEKVYRRS----KRPTNAVVSSIVQVT----------NLPRKRVLKWFEDKRAED-GVPDKRA-
SsHB38-1  : -------RKRFRTKFTAEQKQRMQELSERLGWRLQKRDEAIVDEWCRDI----------GVGKGVFKVWMHNNKHNF---------   PLINC
SsHB77    : -------RKRFRTKFTPEQKERMLEFAEKQGWRIQRNDDGALDRFCDEI----------GVKRQVLKVWMHNHKNQL---------
SsHB87-1  : -------RKRFRTKFTPEQKEQMLAFAERLGWRMQKQDDALVQHFCDQV----------GVRRQVFKVWMHNNKHIG---------
SsHB91-1  : -------RKRFRTKFTAEQKARMLGFAEEAGWRLQKLDDAAVQRFCQEV----------GVKRRVLKVWMHNNKHTL---------
SsHB93-1  : -------RKRFRTKFTPEQKEQMLAFAERLGWRMQKQDDALVQHFCDQV----------GVRRQVFKVWMHNNKHIG---------
SbHB64    : -------RKRFRTKFTPEQKARMLEFAERVGWRLQRLDDGMVQAFCQEI----------GVKRRVLKVWMHNNKHNL---------
AT1G14440 : -------KKRFRTKFTPEQKEKMLSFAEKVGWKIQRQEDCVVQRFCEEI----------GVKRRVLKVWMHNNKIHF---------
SsHB40-1  : MGRPPSTTGAPAFRFLPSEVAEMEARLQQLNN--GIPSRGVLQTLADKFSASPERAGKVAIQPKQVWNWFQNRRYSH-RAKSTRA-   SAWADEE
SsHB44    : MGRPPSTTGAPAFRFLPSEVAEMEARLQQLNN--GIPSRGVLQTLADKFSASPERAGKVAIQPKQVWNWFQNRRYSH-RAKSTRA-
SsHB78-1  : MEGP--------FRFLPEEVKEMEKRLFPVTS--RRLDHILMDELAVKFSYFRGLAGMTPVKSKQVLNWFNNNRNKH-CPKRARE-
SbHB27    : MGRPPSTTGAPAFRFLPSEVAEMEARLQQLNN--GIPSRGVLQTLADKFSASPERAGKVAIQPKQVWNWFQNRRYSH-RAKSTRA-
AT3G18380 : MGRPPS-NGGPAFRFILPEVTEMEAILLQHNT--AMPGRHILEALADKFSESPERKGKVVVQFKQIWNWFQNRRYAL-RARGNKA-
SsHB9     : -------PVRSRWTPKPEQILILESIFNSGM---VNPPKDETVRIRKLLER------FGAVGDANVFYWFQNRRSRS-RRRQRQM-   WOX
SsHB30    : -------PVRSRWTPKPEQILILESIFNSGM---VNPPKDETVRIRKLLER------FGAVGDANVFYWFQNRRSRS-RRRQRQM-
SsHB55-1  : -------DPKPRWNPRPEQIRILEAIFNSGM---INPPRDEIPRIRMRLQE------YGQVGDANVFYWFQNRKARS-KNRLRTT-
SsHB57-1  : -------LANARWNPTKEQVAVLEGLYEHGL---RTPSAEQIQQITGRLRE------HGAIEGKNVFYWFQNHKARQ-RQRQKQD-
SsHB82-1  : -------TARSRWAPKPEQILILESIFNSGM---VNPAKDETARIRRLLER------FGAVRDANVFYWFQNRRSRS-RRRSRQL-
SbHB42    : -------DPKPRWNPRPEQIRILEAIFNSGM---INPPRDEIPRIRMRLQE------YGQVGDANVFYWFQNRKARS-KNKLRTT-
AT1G20710 : -------STRPRWTPTTTQLQILENIYKEGS---GTPNPRRIKEITMELSE------HGQIMEKNVYHWFQNRRARS-KRKQPPT-
                                     L                                                 WF N R
```

Supplementary Figure S3 Sequence alignment of HD domain of representative HB proteins in each subfamily of *S. spontaneum*, *S. bicolor*, and *A. thaliana*
