# Supplementary material for: Expression Profiling and MicroRNA Regulatory Networks of Homeobox Family Genes in Sugarcane Saccharum spontaneum L
Source: Int J Mol Sci. 2022 Aug 5;23(15):8724. doi: 10.3390/ijms23158724 (PMC9369071; doi:10.3390/ijms23158724)
Supplement: Supplementary file 1 [file ijms-23-08724-s001.zip › Supplementary file 8.pdf]

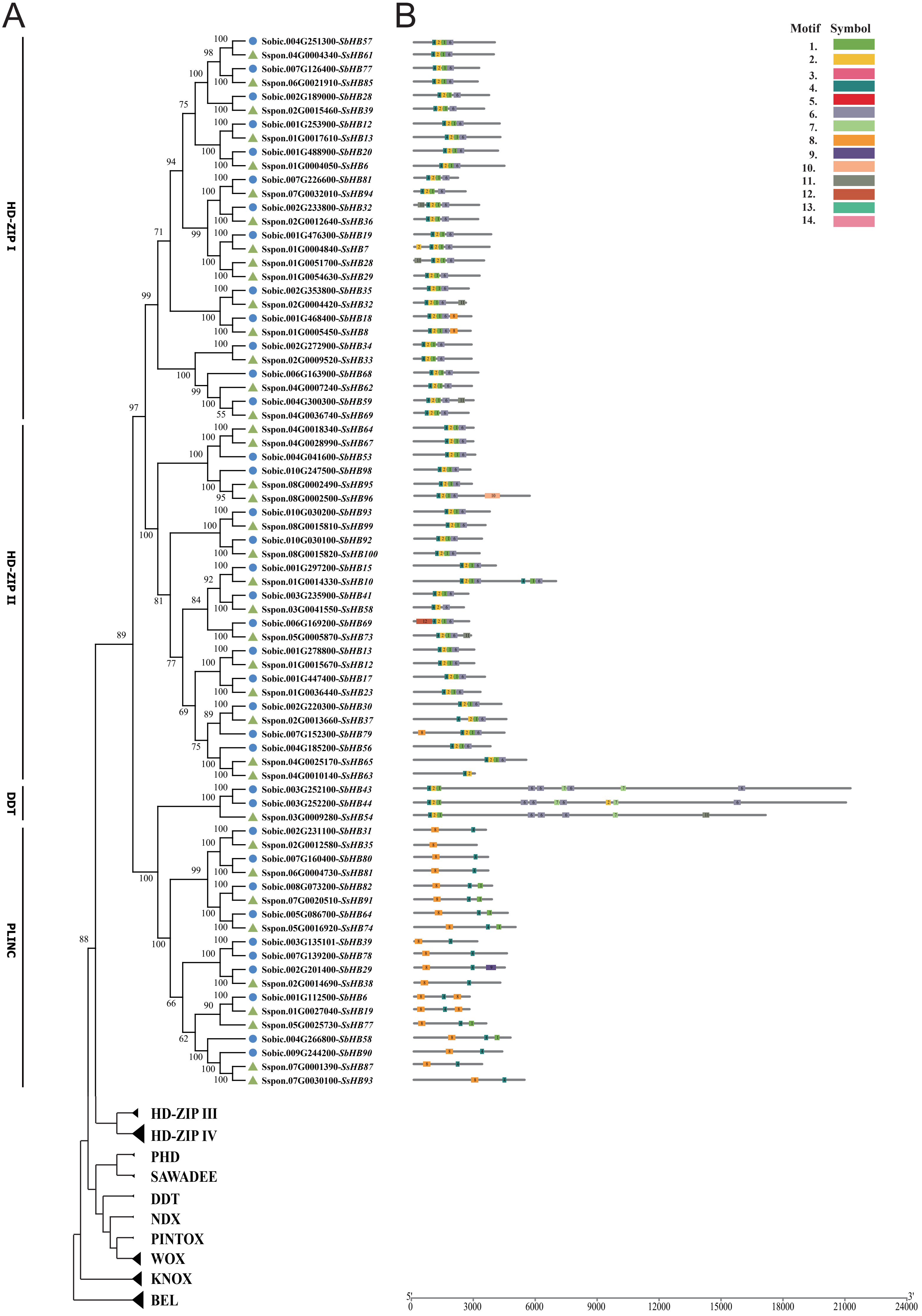
A
B
Motif Symbol
1.
2.
3.
4.
5.
6.
7.
8.
9.
10.
11.
12.
13.
14.
HD-ZIP I
HD-ZIP II
DDT
PLINC
Sobic.004G251300-SbHB57
Sspon.04G0004340-SsHB61
Sobic.007G126400-SbHB77
Sspon.06G0021910-SsHB85
Sobic.002G189000-SbHB28
Sspon.02G0015460-SsHB39
Sobic.001G253900-SbHB12
Sspon.01G0017610-SsHB13
Sobic.001G488900-SbHB20
Sspon.01G0004050-SsHB6
Sobic.007G226600-SbHB81
Sspon.07G0032010-SsHB94
Sobic.002G233800-SbHB32
Sspon.02G0012640-SsHB36
Sobic.001G476300-SbHB19
Sspon.01G0004840-SsHB7
Sspon.01G0051700-SsHB28
Sspon.01G0054630-SsHB29
Sobic.002G353800-SbHB35
Sspon.02G0004420-SsHB32
Sobic.001G468400-SbHB18
Sspon.01G0005450-SsHB8
Sobic.002G272900-SbHB34
Sspon.02G0009520-SsHB33
Sobic.006G163900-SbHB68
Sspon.04G0007240-SsHB62
Sobic.004G300300-SbHB59
Sspon.04G0036740-SsHB69
Sspon.04G0018340-SsHB64
Sspon.04G0028990-SsHB67
Sobic.004G041600-SbHB53
Sobic.010G247500-SbHB98
Sspon.08G0002490-SsHB95
Sspon.08G0002500-SsHB96
Sobic.010G030200-SbHB93
Sspon.08G0015810-SsHB99
Sobic.010G030100-SbHB92
Sspon.08G0015820-SsHB100
Sobic.001G297200-SbHB15
Sspon.01G0014330-SsHB10
Sobic.003G235900-SbHB41
Sspon.03G0041550-SsHB58
Sobic.006G169200-SbHB69
Sspon.05G0005870-SsHB73
Sobic.001G278800-SbHB13
Sspon.01G0015670-SsHB12
Sobic.001G447400-SbHB17
Sspon.01G0036440-SsHB23
Sobic.002G220300-SbHB30
Sspon.02G0013660-SsHB37
Sobic.007G152300-SbHB79
Sobic.004G185200-SbHB56
Sspon.04G0025170-SsHB65
Sspon.04G0010140-SsHB63
Sobic.003G252100-SbHB43
Sobic.003G252200-SbHB44
Sspon.03G0009280-SsHB54
Sobic.002G231100-SbHB31
Sspon.02G0012580-SsHB35
Sobic.007G160400-SbHB80
Sspon.06G0004730-SsHB81
Sobic.008G073200-SbHB82
Sspon.07G0020510-SsHB91
Sobic.005G086700-SbHB64
Sspon.05G0016920-SsHB74
Sobic.003G135101-SbHB39
Sobic.007G139200-SbHB78
Sobic.002G201400-SbHB29
Sspon.02G0014690-SsHB38
Sobic.001G112500-SbHB6
Sspon.01G0027040-SsHB19
Sspon.05G0025730-SsHB77
Sobic.004G266800-SbHB58
Sobic.009G244200-SbHB90
Sspon.07G0001390-SsHB87
Sspon.07G0030100-SsHB93
HD-ZIP III
HD-ZIP IV
PHD
SAWADEE
DDT
NDX
PINTOX
WOX
KNOX
BEL
5'
3'
0
3000
6000
9000
12000
15000
18000
21000
24000

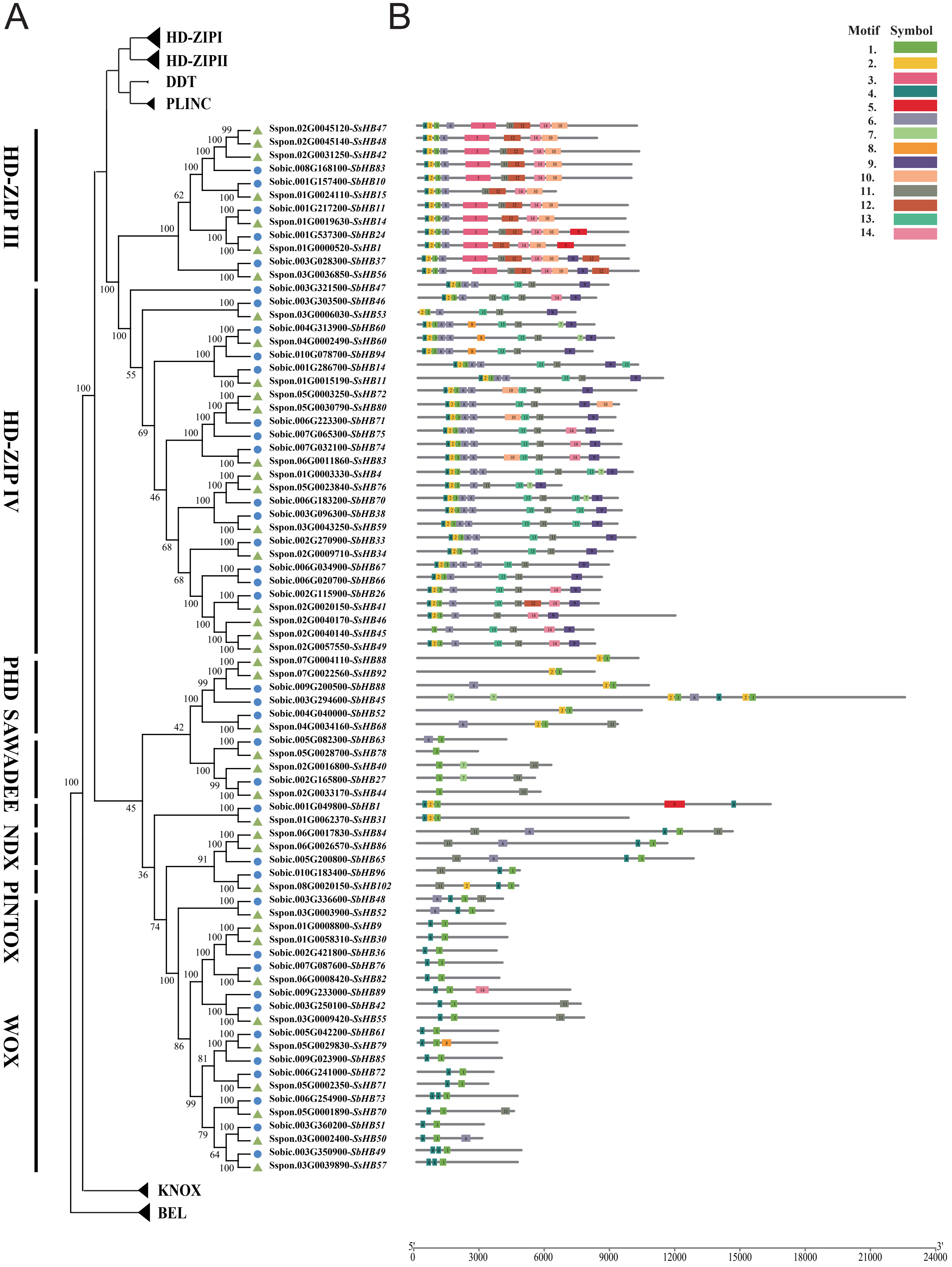
A
B
HD-ZIPI
HD-ZIPII
DDT
PLINC
HD-ZIP III
HD-ZIP IV
PHD SAWADEE NDX PINTOX
WOX
KNOX
BEL
Sspon.02G0045120-SsHB47
Sspon.02G0045140-SsHB48
Sspon.02G0031250-SsHB42
Sobic.008G168100-SbHB83
Sobic.001G157400-SbHB10
Sspon.01G0024110-SsHB15
Sobic.001G217200-SbHB11
Sspon.01G0019630-SsHB14
Sobic.001G537300-SbHB24
Sspon.01G0000520-SsHB1
Sobic.003G028300-SbHB37
Sspon.03G0036850-SsHB56
Sobic.003G321500-SbHB47
Sobic.003G303500-SbHB46
Sspon.03G0006030-SsHB53
Sobic.004G313900-SbHB60
Sspon.04G0002490-SsHB60
Sobic.010G078700-SbHB94
Sobic.001G286700-SbHB14
Sspon.01G0015190-SsHB11
Sspon.05G0003250-SsHB72
Sspon.05G0030790-SsHB80
Sobic.006G223300-SbHB71
Sobic.007G065300-SbHB75
Sobic.007G032100-SbHB74
Sspon.06G0011860-SsHB83
Sspon.01G0003330-SsHB4
Sspon.05G0023840-SsHB76
Sobic.006G183200-SbHB70
Sobic.003G096300-SbHB38
Sspon.03G0043250-SsHB59
Sobic.002G270900-SbHB33
Sspon.02G0009710-SsHB34
Sobic.006G034900-SbHB67
Sobic.006G020700-SbHB66
Sobic.002G115900-SbHB26
Sspon.02G0020150-SsHB41
Sspon.02G0040170-SsHB46
Sspon.02G0040140-SsHB45
Sspon.02G0057550-SsHB49
Sspon.07G0004110-SsHB88
Sspon.07G0022560-SsHB92
Sobic.009G200500-SbHB88
Sobic.003G294600-SbHB45
Sobic.004G040000-SbHB52
Sspon.04G0034160-SsHB68
Sobic.005G082300-SbHB63
Sspon.05G0028700-SsHB78
Sspon.02G0016800-SsHB40
Sobic.002G165800-SbHB27
Sspon.02G0033170-SsHB44
Sobic.001G049800-SbHB1
Sspon.01G0062370-SsHB31
Sspon.06G0017830-SsHB84
Sspon.06G0026570-SsHB86
Sobic.005G200800-SbHB65
Sobic.010G183400-SbHB96
Sspon.08G0020150-SsHB102
Sobic.003G336600-SbHB48
Sspon.03G0003900-SsHB52
Sspon.01G0008800-SsHB9
Sspon.01G0058310-SsHB30
Sobic.002G421800-SbHB36
Sobic.007G087600-SbHB76
Sspon.06G0008420-SsHB82
Sobic.009G233000-SbHB89
Sobic.003G250100-SbHB42
Sspon.03G0009420-SsHB55
Sobic.005G042200-SbHB61
Sspon.05G0029830-SsHB79
Sobic.009G023900-SbHB85
Sobic.006G241000-SbHB72
Sspon.05G0002350-SsHB71
Sobic.006G254900-SbHB73
Sspon.05G0001890-SsHB70
Sobic.003G360200-SbHB51
Sspon.03G0002400-SsHB50
Sobic.003G350900-SbHB49
Sspon.03G0039890-SsHB57
Motif Symbol
1.
2.
3.
4.
5.
6.
7.
8.
9.
10.
11.
12.
13.
14.
5'
3'
0
3000
6000
9000
12000
15000
18000
21000
24000

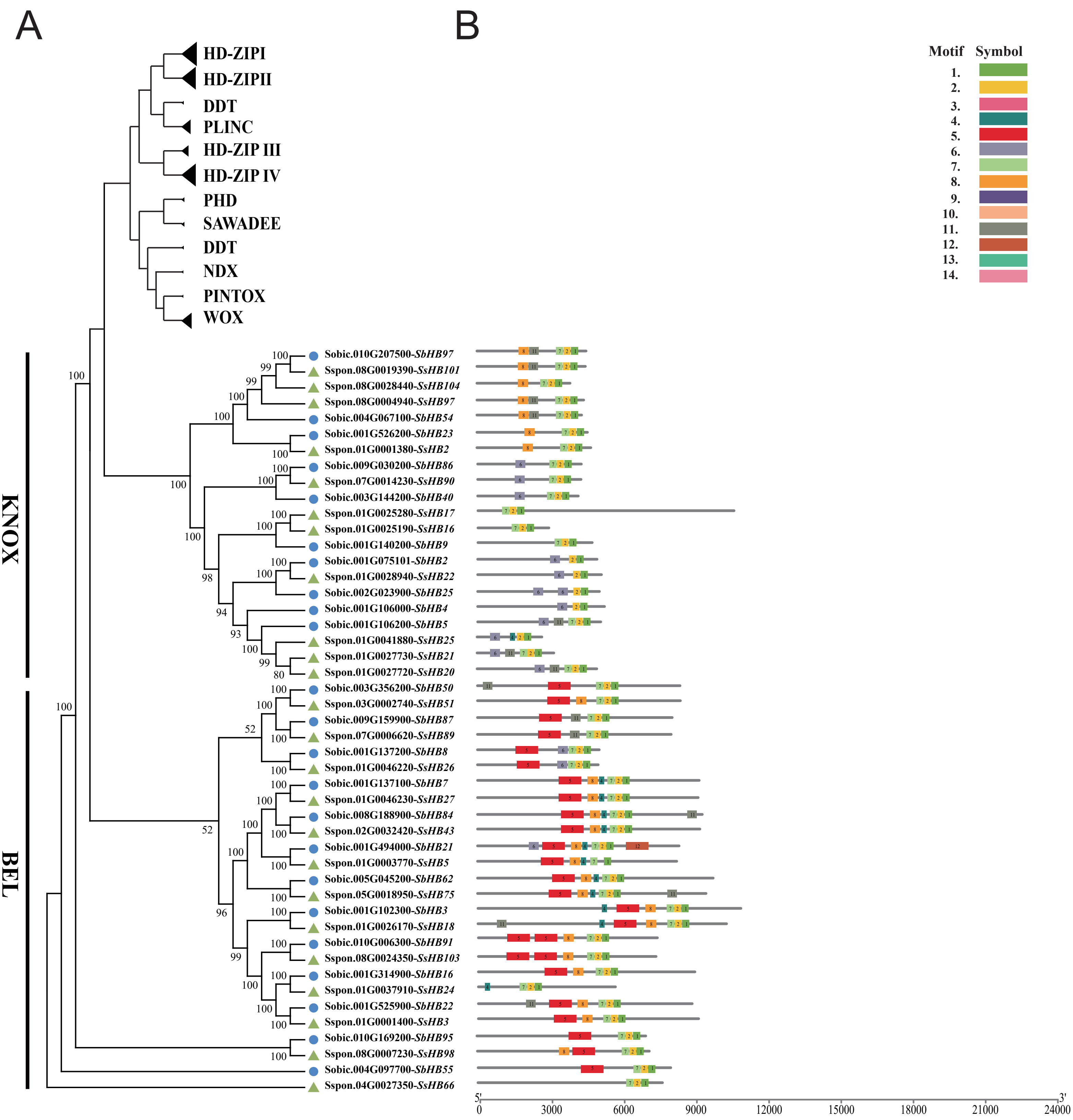

Supplementary Figure S5 Architecture of conserved motifs in HBs between *S. spontaneum* and *S. bicolor.* (A) The ML tree was generated in iq-tree with 1000 bootstrap replicates. (B) Motif analysis of SsHBs and SbHBs. The numbers 1-14 of motifs are displayed in different color boxes
