# Supplementary figures and images for: Expression Profiling and MicroRNA Regulatory Networks of Homeobox Family Genes in Sugarcane Saccharum spontaneum L
Source: Int J Mol Sci. 2022 Aug 5;23(15):8724. doi: 10.3390/ijms23158724 (PMC9369071; doi:10.3390/ijms23158724)

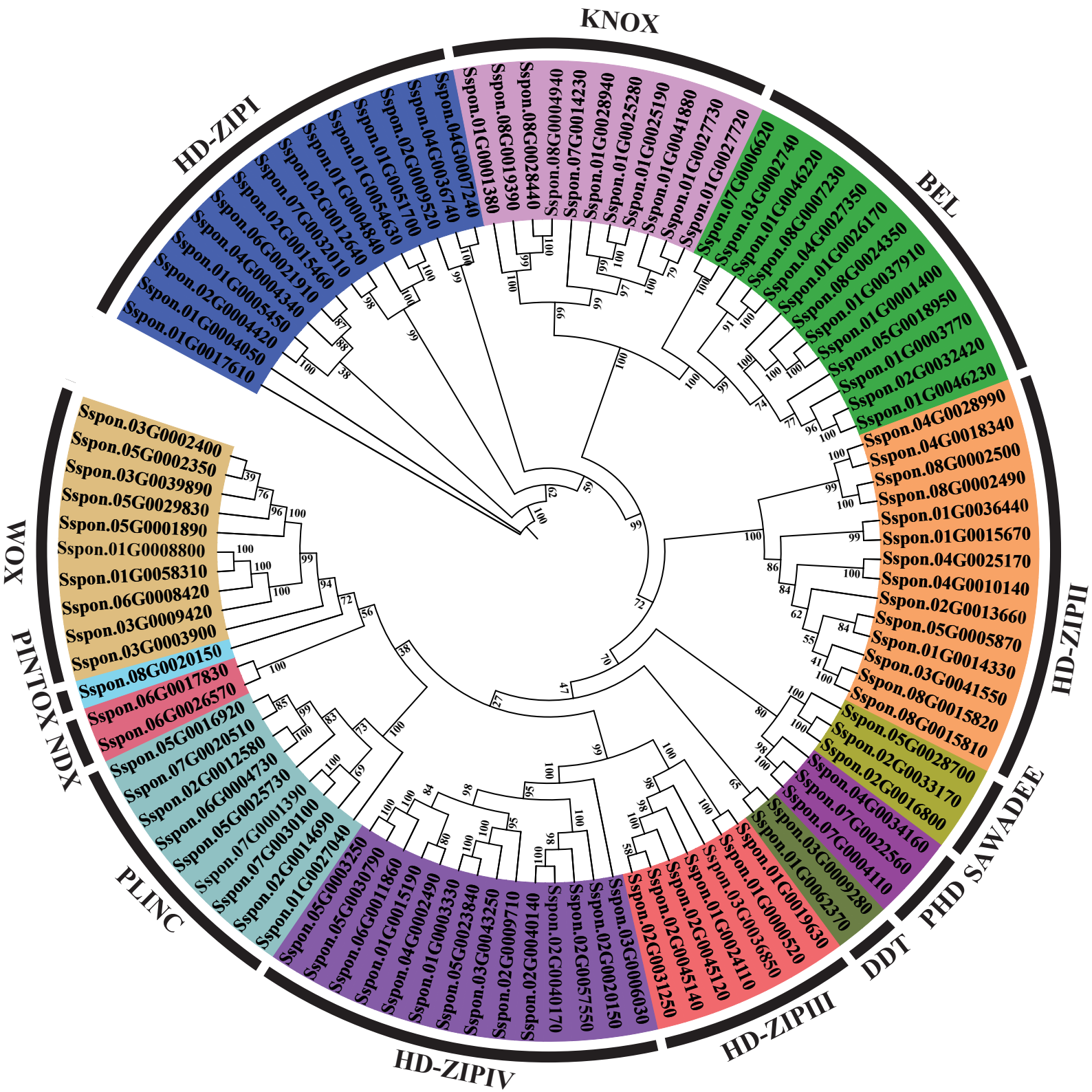

Supplementary Figure S1 The unrooted ML tree of SsHBs

Supplement: Supplementary file 1 [file ijms-23-08724-s001.zip › Supplementary file 4.pdf]

A

HD-ZIP I

HD-ZIP II

DDT

PLINC

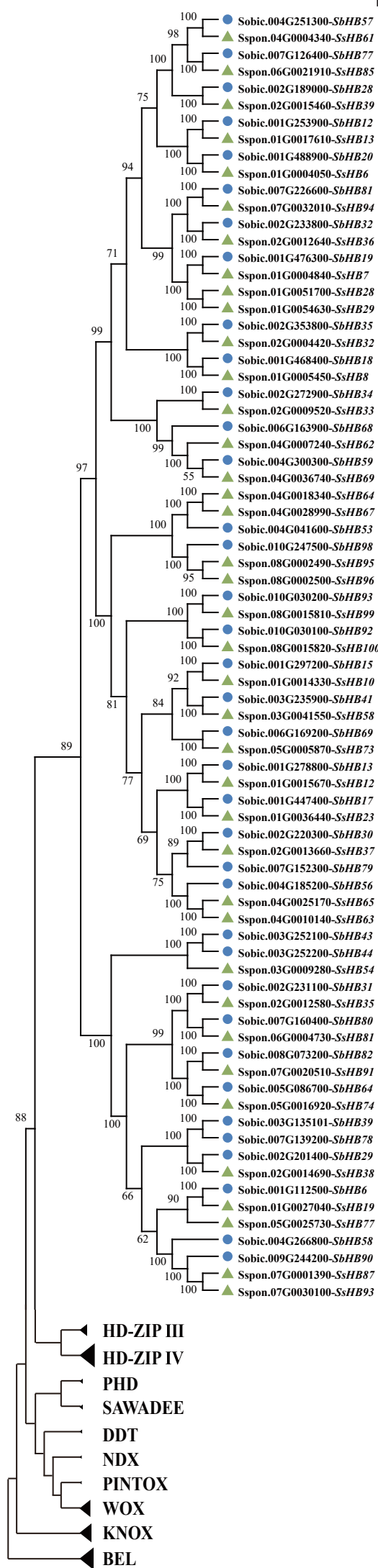

B

UTR CDS

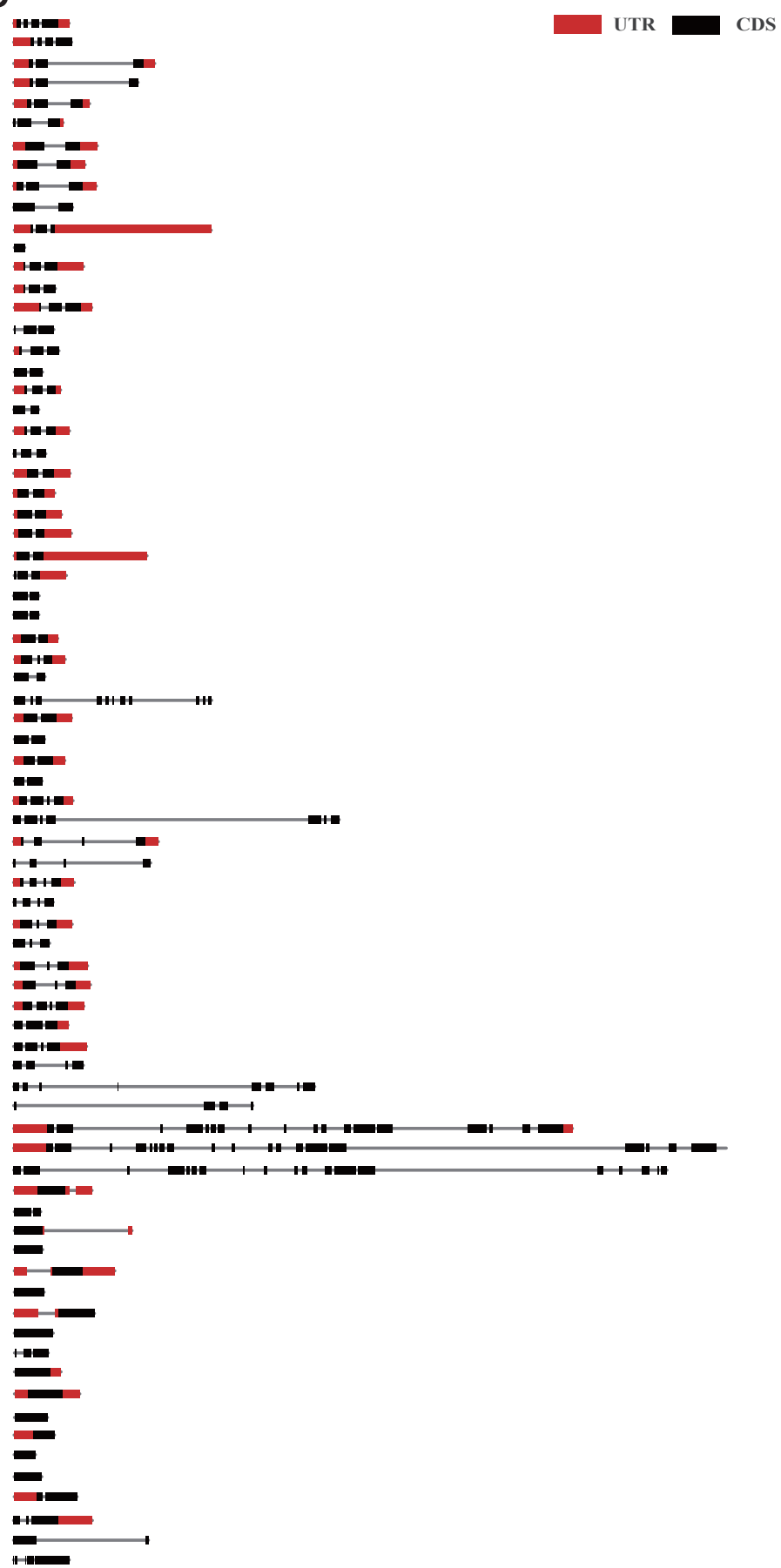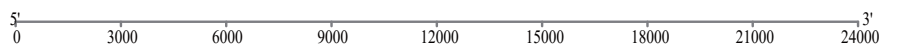

A

HD-ZIP III

HD-ZIP IV

PHD SAWADEE NDX PINTOX

WOX

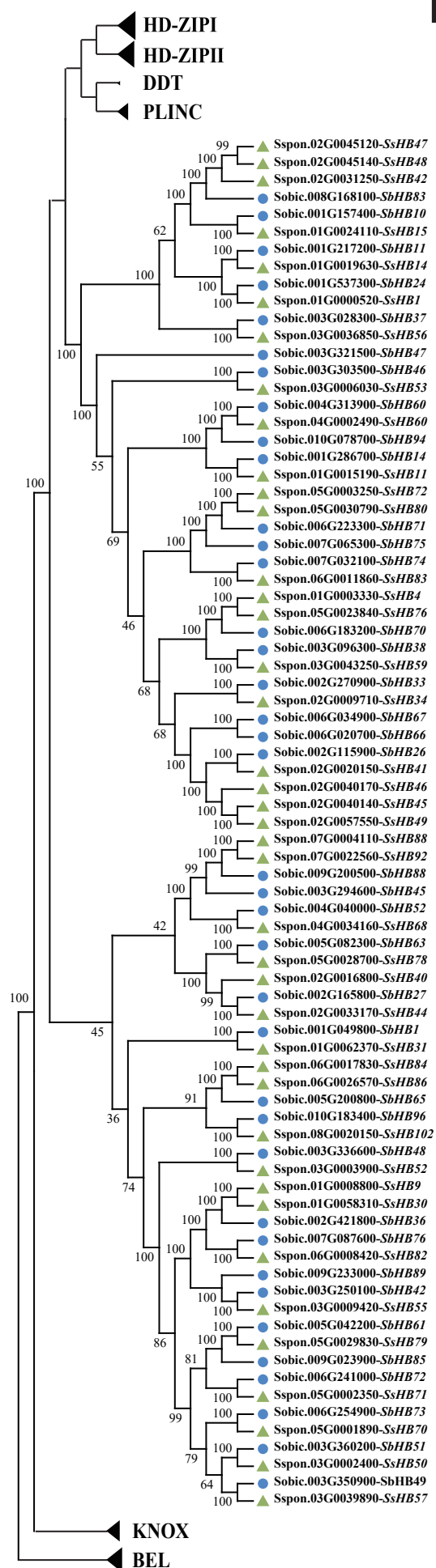

B

UTR CDS

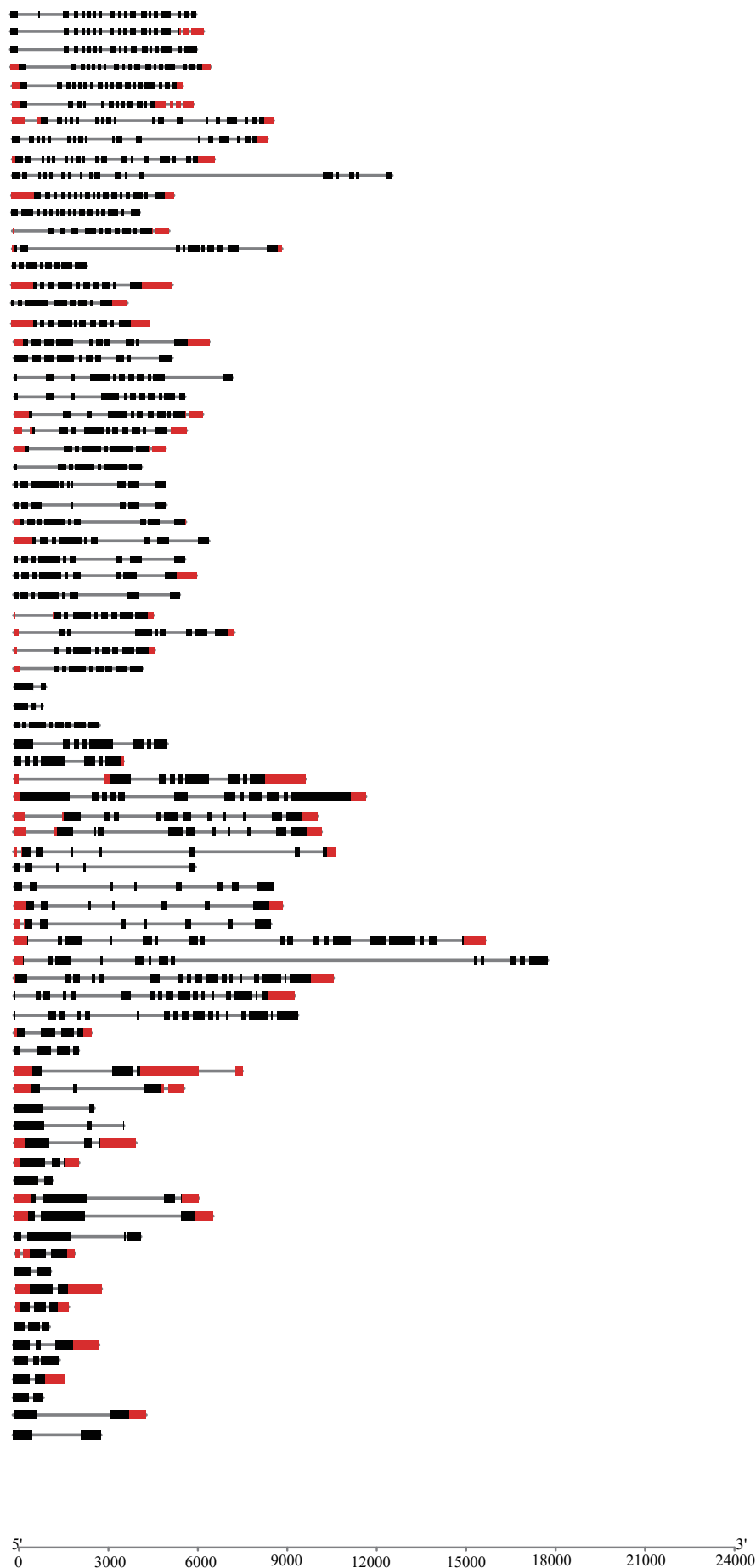

# B

# B

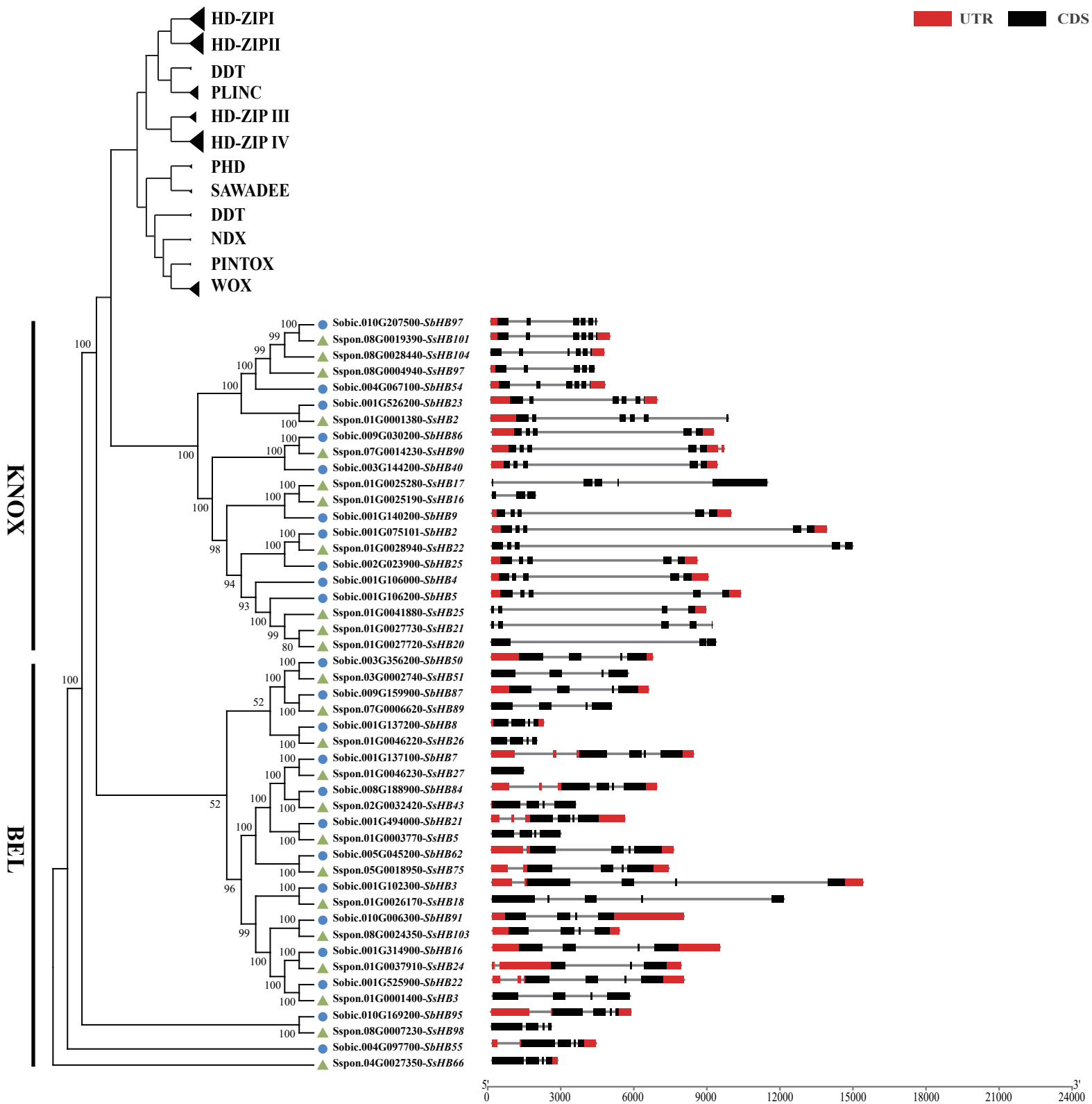

Supplement: Supplementary file 1 [file ijms-23-08724-s001.zip › Supplementary file 7.pdf]
